# "You are always left with doubts": information access among HPV-positive women in Greater Buenos Aires

**DOI:** 10.15446/rsap.V25n2.102896

**Published:** 2023-03-01

**Authors:** Lucila Szwarc, Victoria Sánchez-Antelo, Melisa Paolino, Silvina Arrossi

**Affiliations:** 1 LS: Socióloga. M.Sc. Genre, Politique et Sexualité. Ph.D. Ciencias Sociales. Centro de Estudios de Estado y Sociedad (CEDES)/Consejo Nacional de Investigaciones Científicas y Técnicas (CONICET). Buenos Aires, Argentina. lucilaszwarc@gmail.com Centro de Estudios de Estado y Sociedad (CEDES)/Consejo Nacional de Investigaciones Científicas y Técnicas (CONICET) Buenos Aires Argentina; 2 VS: Socióloga. M.Sc. Empleo y Política Social. Ph.D. Ciencias Sociales. Centro de Estudios de Estado y Sociedad (CEDES)/Consejo Nacional de Investigaciones Científicas y Técnicas (CONICET). Buenos Aires, Argentina. vsanchezantelo@gmail.com Ciencias Sociales. Centro de Estudios de Estado y Sociedad (CEDES)/Consejo Nacional de Investigaciones Científicas y Técnicas (CONICET) Buenos Aires Argentina; 3 MP: Socióloga. Ph.D. Ciencias Sociales. Esp. Salud Pública. Centro de Estudios de Estado y Sociedad (CEDES)/Consejo Nacional de Investigaciones Científicas y Técnicas (CONICET). Buenos Aires, Argentina. melisa@cedes.org Centro de Estudios de Estado y Sociedad (CEDES)/Consejo Nacional de Investigaciones Científicas y Técnicas (CONICET) Buenos Aires Argentina melisa@cedes.org; 4 SA: Socióloga. M.Sc. Public Health. Ph.D. Demografía. Centro de Estudios de Estado y Sociedad (CEDES)/Consejo Nacional de Investigaciones Científicas y Técnicas (CONICET). Buenos Aires, Argentina. silviarrossi2020@gmail.com Centro de Estudios de Estado y Sociedad (CEDES)/Consejo Nacional de Investigaciones Científicas y Técnicas (CONICET) Buenos Aires Argentina

**Keywords:** Human papillomavirus, screening, preventive health services, access to information, communication barriers *(source: MeSH, NLM)*, Papillomavirus humano, tamizaje, servicios preventivos de salud, acceso a la información, barreras de comunicación *(fuente: DeCS, BIREME)*

## Abstract

**Objective:**

Women who are Human Papillomavirus (HPV) positive with a normal Pap smear (HPV+/normal Pap) present a high risk of developing high-grade lesions but they exhibit very low levels of adherence in Argentina. This study was aimed to identify the information needs, sources of information, and barriers to accessing information among HPV-positive women with normal Pap after receiving their screening results.

**Methods:**

We conducted 22 semi-structured virtual interviews with HPV-positive women with normal Pap (HPV+/normal Pap) women using a qualitative research strategy. Eligible women were between 30 and 64 years old and resided in a suburban Buenos Aires area. The interview data was thematically analyzed using the modules of the interview guide. Emergent categories were coded as subthemes, enabling a thematic analysis.

**Results:**

Perceived information needs among HPV-positive women with normal Pap smears include understanding: the sexual transmission of the virus (including dismantling the association with infidelity and considering non-sexual transmission possibilities), the meaning of the diagnosis in relation to cancer, and the follow-up procedures despite the absence of treatment. On the other hand, unperceived information needs encompass recognizing the association between an HPV+ result and other gynecological health problems, as well as dispelling socio-cultural misconceptions about HPV (e.g., the misconception that high-risk HPV only affects young women with multiple sexual partners). Common sources of information for these women include medical visits, internet resources, and advice from close relatives. However, barriers to accessing information include encountering inaccurate online information, receiving insufficient information from healthcare professionals, and facing difficulties in raising questions and concerns during medical consultations.

**Conclusions:**

It is crucial to strengthen the processes for delivering results during the medical consultation. New formats to provide information to HPV-positive women, both with normal and abnormal Pap smear, should also be considered.

## RESUMEN

**Objetivos:**

Las mujeres con resultado positivo de test de Virus de Papiloma Humano (VPH) y resultados negativo o normal de Papanicolau (VPH+/Pap-) presentan un riesgo alto de desarrollar lesiones en el cuello del útero, pero tienen muy bajos niveles de adherencia al seguimiento en Argentina. Este estudio se propuso identificar las necesidades de información, las fuentes de información y las barreras de acceso a la información de mujeres con VPH positivo y Pap normal o negativo luego de haber recibido los resultados del tamizaje.

**Métodos:**

Con una estrategia de investigación cualitativa, se realizaron 22 entrevistas virtuales semiestructuradas a mujeres con VPH positivo y Papanicolaou normal o negativo (VPH+/Pap-). Las mujeres elegibles tenían entre 30 y 64 años y residían en un área suburbana de Buenos Aires. Los datos de las entrevistas fueron analizados temáticamente utilizando los módulos de la guía de entrevistas. Se codificaron las categorías emergentes como subtemas, lo cual posibilitó un análisis temático.

**Resultados:**

Existen necesidades de información percibidas entre las mujeres con VPH positivo y Papanicolaou normal o negativo. Estas necesidades incluyen información sobre: la transmisión sexual del virus (desmontando la asociación con la infidelidad y considerando la posibilidad de transmisión no sexual), el significado del diagnóstico y su relación con el cáncer, así como el seguimiento y la ausencia de tratamiento. Por otro lado, existen necesidades de información no percibidas, como la asociación entre el resultado y otros problemas de salud ginecológicos, y las ideas erróneas socioculturales sobre el VPH. Las fuentes de información comunes para estas mujeres son la visita médica, la consulta en internet y el diálogo con los familiares. Sin embargo, existen barreras que dificultan el acceso a la información, como la información inexacta en internet, la falta de información proporcionada por los profesionales y las dificultades para hacer preguntas durante la consulta médica.

**Conclusiones:**

Es fundamental reforzar los procesos de entrega de resultados y considerar nuevos formatos para proporcionar información a las mujeres VPH+, tanto con Pap normal como anormal.

The efficacy of the Human Papillomavirus (HPV) test as a primary screening for cervical cancer (CC) prevention has been widely proven 1,2. For its implementation to be cost-effective, women's adherence to follow-up is crucial. Evidence has shown that in addition to socio-economic conditions, factors related to health care organization, such as the quality of communication and care during the delivery of results 3-5, and individual aspects, such as fears and beliefs, play key roles in women's adherence to screening and follow-up 6,7. Poor or insufficient interaction with providers may lead to misinterpretation of abnormal Pap or positive HPV test results 8,9, increased levels of stress and anxiety 10,11, or reluctance to seek proper follow-up 5,12. Studies have also found that non-adherent women often have poor knowledge or misconceptions about the need for follow-up, incorrect beliefs about the meaning of screening results, fear of cancer, and a low perception of risk 3,13,14. Conversely, women who reported having had the opportunity to ask questions or received clear explanations about their results and subsequent steps showed higher adherence to treatment 15,16.

The HPV test establishes a diagnostic entity with ambiguous connotations, which requires women to process complex concepts: it is an asymptomatic, sexually transmitted virus with follow-up indication, that can either disappear, or remain in the body, eventually producing precancerous lesions. HPV-positive women often overestimate its seriousness, do not understand its ambiguity, express confusion about the sexual trans-mission of the virus or confuse the test with the Pap 17,18. This difficulty to understand HPV positivity may be greater for women who are HPV-positive with a normal triage Pap 9. If the triage Pap is normal, HPV-positive results indicate the presence of a virus that has no manifestation, making its understanding more complex. The recommendation to repeat the test after 18 months 19 may also accentuate the feelings of ambiguity and uncertainty that women may express when receiving screening results 20,21.

Lack of understanding and knowledge among women can be conceptualized as "information needs", which in relation to HPV positivity refers to women's doubts or confusions about the meaning of the result and its implications 22,24. Communication and understanding problems during the delivery of results may function as information barriers. A research focusing on HPV-positive/abnormal Pap women conducted in Ireland, found that information overload during a single medical consultation, together with women's perceptions of the health care system and concerns around the result, may function as barriers to information access 23. To overcome information barriers in medical consultation, women may look for answers on the internet or during informal conversations 24,25, which may expose them to inaccurate and false data. However, barriers to information access have been poorly studied 23; and to our knowledge, information needs and sources of HPV-positive/normal Pap women have not been specifically analyzed.

We aimed to identify the needs, sources, and barriers to access information of HPV-positive/normal Pap women in Greater Buenos Aires; evidence produced by this study will allow to respond to a knowledge gap and contribute to develop communication strategies for this specific group of women.

## MATERIAL AND METHODS

### Setting

The study took place in Ituzaingó, Greater Buenos Aires, with a population of 167,000 inhabitants, 54 % of whom are female 26. The local public health system includes a tertiary referral hospital, 5 primary health-care centers, 1 primary sexual and reproductive health-care center, and 3 specialized care centers. 35 % of Ituzaingó's target population for HPV testing (women aged 30-64) have public health insurance exclusively and receive health care free of charge.

Primary HPV screening has been offered free of cost since 2017 in all public health care facilities, for women aged 30 years and older. HPV testing and cytology triage are collected simultaneously, but cytology is read only if the HPV test is positive. Women who have HPV-positive and normal cytology are recommended re-screening in 18 months. Individuals whose samples are classified as ASCUS or worse are referred to colposcopy and biopsy if needed. Women who are HPV negative are recommended re-screening in 5 years 19.

### Data collection

We used a qualitative research strategy focused on people's perception of reality 27. We analyzed the information needs and sources, and barriers to access information about an HPV-positive result, from women's experiences and perspectives.

Fieldwork took place between July and December 2020. We performed 22 semi-structured interviews with women aged 30-64, who had performed HPV testing in a public health service in Ituzaingó in 2019, in the last 12 months before the interviews, and had HPV+/normal Pap results. The 40' interviews were conducted through Zoom platform and were digitally recorded and transcribed later on. The number of interviews responded to theoretical saturation criteria (saturation occurs when no new thematic axes are identified).

We used a convenience sample. Contacts with interviewees were established through the staff of Ituzaingó Health Secretary. Initially, staff from the public health center contacted women asking whether they would accept to participate. If they agreed, a researcher would make a call explaining the objectives of the study and inviting women to take part in a virtual interview.

After the interviews, we provided women with accurate information and answered their questions. The information was collected in Spanish by a female social sciences researcher, who was not related to the health care institutions or their authorities.

We used a semi-structured guide for semi-structured interviews organized in three modules: a) Women's information needs, understood as those aspects linked to the meaning of the result and its implications about which women express doubts or confusion. We distinguish between perceived information needs -the information needs which women perceive as such-, and unperceived information needs -aspects that women consider themselves to understand, but about which they express significant confusion or inaccurate understanding-; b) The needs in terms of the sources of information consulted and preferred; c) Barriers to access information.

### Analytic Approach

Interviews data was analyzed thematically using the modules of the interviews guide. We coded emergent categories as subthemes, allowing a thematic analysis 28 of the discussions regarding women's perceptions about information needs, barriers to access information and sources of information. Transcripts were analyzed independently by two researchers, to later compare, debate, and resolve the inconsistencies with the other members of the team.

### Ethical aspects

The study's protocol was approved by the Ethics Committee of the Gino Germani Research Institute (UBA). Women had to agree orally to an informed consent. The anonymity of participants was guaranteed. Procedures were followed to guarantee conformity with the principles and ethical standards of the 1975 Declaration of Helsinki and its subsequent revisions and with Resolution 8430 (1993), of the Colombian Ministry of Health.

## RESULTS

### Characteristics of the interviewed women

Table 1 shows the characteristics of the interviewed women (n=22).

### Perceived information needs

In order of recurrence, in the first place, interviewed women needed more information about the sexual trans-mission of the virus. Many of them did not understand how they could have contracted it. If they were in a relationship, they tended to associate HPV positivity with an infidelity of their partners. Faced with the concern of an infidelity, it was common for them to report relief when informed that the sexual transmission may have occurred long ago, or that the virus could have been acquired by non-sexual means. Finally, women had questions about the possibility of infecting others.

[...] I say: "How did I get it, [...] I have had a partner for...", at that time it had been seven years, and I never cheated on him. [...] and he swears to me that he neither [...] (I14).

Secondly, women expressed doubts about the meaning of the diagnosis. They asked about its general meaning, or specifically about the link between the virus and cancer. This caused them concern because they assumed HPV was a serious disease.

I was seen first by a doctor, and she told me that if it came out positive, it was dangerous. [...] I asked her: "Does this mean that I have cancer?" (I20).

Thirdly, women had doubts about follow-up, treatment and lack of treatment. They asked whether the virus had a cure, or why practitioners did not mention procedures they had heard about, such as biopsies or conizations.

Q: And what are the questions you left unasked at the time? A: If there was any medicine available or anything to do, what could I do, is there any kind of vaginal suppository... (I16) "I told him [the doctor]: 'Why don't you send me for a biopsy?" (I13).

### Unperceived information needs

It was common for women to perceive other gynecological problems (such as heavy menstruation, cysts, etc.) as being linked to the result.

I was already in pain, and I had a lot of hemorrhages, it is as if after receiving that result, [...] I couldn't stop thinking about it, and the pain became worse (I7).

Women expressed ideas about HPV, linked to previous social beliefs, which increased their misunderstandings. They mentioned that it was necessary to inform younger women about HPV because, according to them, they were the ones who should "get checked" the most (Interviews 1, 8 and 14). However, local guidelines recommend HPV testing for women aged 30 or older 19. To a lesser extent, a woman confused the concept of "latent virus" and referred that "we all have cancer; cancer is asleep until it wakes up" (Interview 25).

### Preferred sources and information support

The medical consultation was the information source women valued the most. However, they referred that the information provided by practitioners was usually insufficient, which made them look for more by their own means.

[...] I knew what the doctor had told me, and if I had any doubts, I would search a little bit more [...]. You are always left with doubts, maybe they tell you and [...] you understand it and then you say "I didn't understand this, I'm going to see what it's about" [...] (I19).

The second most consulted source was the internet. Women mentioned Google searches that led to official and unofficial organization webpages and YouTube videos. They also reported forums and social network groups for people with similar conditions. Finally, the interviewees consulted organizations or personalities' official social network accounts.

[...] later, through Argentine League for the Fight Against Cancer (LALCEC) website, I started to get information, because they [health practitioners] only gave me the results and they told me that I had to come back after a year and a half (I15).

Some women reported that they sought the internet trying to find appropriate answers differentiating accurate information from that one that it was not. For this purpose, they consulted official institutions and professionals' websites and compared information found there with data from other websites. In health forums and groups, they paid attention to other users' comments. When possible, they sought to validate what they have found by asking doctors.

Q: And when you find information on the internet or in an app, how do you know if the information is reliable? A: [...] because of people's comments: "Don't believe it, it's all lies". [...] "Do not use this site". You always read the comments (I2).

Q: [...] how do you know that the information on the internet is reliable?

A: I don't know, I can't be sure, but I can compare it. So... when I go to a real consultation with a doctor, I ask him all those things I looked up [...] (I3).

It was also common for women to receive information from people close to them, generally women who have had similar results, that helped them complete the information they received at the medical appointment.

[...] But afterwards I talked to other girls, and the same thing had happened to one of them, but since she had already had a family history, they did a biopsy to her, [...] and I told it to the gynecologist when I went last month (I13).

### Barriers to information access

The primary and principal barrier to accessing information pertains to doctor-patient communication. Women perceive that healthcare professionals employ complex terminology, fail to provide comprehensive information, or do not allocate sufficient time to offer explanations.

[...] Integral Health Care Program for retirees and pensioners (PAMI) doctors have an eye on the watch. They give each patient a certain amount of time, you don't have time to keep chatting and asking [...] (I23).

[...] They don't give you much information, they tell you just what's necessary, it's like they don't want to worry you too much, but they don't leave you with any peace of mind either. [...] (I6).

Women also expressed difficulties in asking questions during the consultation.

It's usual, when you go to see the doctor, that after leaving the consultation you think: "Oh! Why didn't I ask him this, I had so many questions to ask him, and I just didn't ask him this", and time goes by, and doubts remain (I22).

Another information barrier reported by women was the presence of fake or inaccurate information on the internet.

I used to surf the internet, but I found a lot of wrong information and many things that weren't real, and I got scared (I8).

## DISCUSSION

Our findings indicate that HPV+/normal Pap women faced several problems to access adequate information. First, although women would have preferred that the medical consultation was the main information source, a main barrier to this was the lack of explanations provided by practitioners or the insufficient time given to women to ask questions. Left with doubts, to clearly understand the meaning of the test results, it was usual for women to look for more data on the internet or to talk to close relatives. A main issue was how to distinguish between accurate and false data on the internet, therefore, they tried, when possible, to validate what they had found by asking doctors. Despite consulting several sources, interviewees still expressed unsolved information needs.

Evidence shows that HPV-positive women have unsolved information needs about the sexual transmission of the virus, the meaning of the result and its relation to cancer, as well as aspects related to follow-up and treatment 22,24,29. Although in our study these information needs were present, doubts about sexual transmission and lack of treatment stood out over those about the link between HPV and cancer. This could be due to the focus that practitioners may be placing when delivering results to HPV+/normal Pap women, in comparison to women with abnormal Pap results, given that immediate clinical intervention is not required in those cases. Studies had shown that women mainly relate abnormal Pap results to the possibility of having pre-cancer or cancer, which causes them fear and concern 30,31.

Another contribution from our research is the unperceived information needs of HPV+/normal Pap women, mainly related to the screening age, and the symptoms of the HPV infection. A prior study conducted in Jujuy, northern Argentina, in 2016, found that HPV-positive women, both with normal and abnormal Pap smears, had different non-perceived information needs than the ones found in this study, mainly doubts about diagnostic procedures and treatments and their effects on the body 9. In our research, focused exclusively on HPV+/ normal Pap women, this aspect did not emerge. Our interviewees tended to relate a variety of gynecological symptoms, such as heavy or painful menstruations, to the diagnosis they had received. A research conducted in Sweden described that women who continued the follow-up process after an abnormal Pap result reconceptualized the way they looked at their bodies, as the cervix became perceived and felt, first through the mediation of the doctor's words, and then through the manipulation in biopsy and treatment 32. In our research, since there was neither a manifestation of the virus nor medical intervention, women, perhaps looking for signs in their bodies, tended to associate previous gynecological symptoms to the result, even though they were not, in fact, related to it. Interviewees also emphasized the importance of informing and testing younger women, which showed their lack of knowledge about the recommended target ages for screening (from the age of 30) 19. According to other studies, women may associate the risk of contracting HPV with a sexually active life and having multiple sexual partners, so they identify younger women to be at higher risk, 33,34 in line with a widespread consideration of the medical community. In Latin America countries, many practitioners recommend screening from the onset of sexual intercourse, regardless of age, irrespective of national guidelines 35.

Our study showed that although women stated they chose to obtain information during the medical consultation, when health practitioners did not answer their information's needs, they sought it by other means, such as the internet or talking to people they were close to. Similar results were reported in other countries 24,25. Even if interviewees tried to resort to official sources and to registered professionals online, these sources expose them to inaccurate information, as a study about exposure, access and uses regarding health information suggest 36.

The main barrier to access information referred to doctor-patient communication. According to women, practitioners do not devote enough time to deliver HPV-positive results or they use a difficult or technical vocabulary, as a study carried out in Mexico also pointed out 18. Other Latin American studies showed that health systems may not provide exhaustive or clear information about a Pap test result 37,38 or may not offer an adequate caring behavior during consultation 39. In other contexts, women reported having received insufficient information or having difficulties understanding practitioner's indications and vocabulary, regarding an HPV-positive test and/or an abnormal Pap result 5,40. Finally, women mentioned their own difficulties in asking questions during the medical appointment. According to a study conducted in Ireland, information overload can turn into a barrier for women to access information 23. This concerns doctor-patient communication and the lack of dialogues based on listening, support, and an accessible and understandable language. Unsolved information needs about an HPV-positive test may increase stress and anxiety levels and be a barrier for women to understand the result and subsequent need for follow-up. Our study suggests that innovations to improve health providers' communication and increase women's understandings about HPV-positive results are needed. Also, information available online and the possibility to offer communication and support after the delivery of results should be addressed, in order to respond to women's needs and reduce access barriers to follow-up.

This study has some limitations. Results may be subject to bias and disproportionately reflect opinions from participants who had particular experiences, given the non-probabilistic sample. Another possible limitation is that the generalizability of our findings may be limited due to the small sample size and the specific study setting; however, we considered them sufficient for qualitative research, because clear themes emerged from the analysis ♣
